# Supplementary material for: Vulnerability of smallholder farmers to climate variability and change across different agro-ecological Zones in Oromo Nationality Administration (ONA), North east Ethiopia
Source: PLoS One. 2022 Jun 22;17(6):e0268094. doi: 10.1371/journal.pone.0268094 (PMC9216564; doi:10.1371/journal.pone.0268094)
Supplement: S1 File — (DOCX) [file pone.0268094.s002.docx]

Household Survey

Survey on Agroecological Based Smallholder Farmers’ Livelihood Vulnerability to Climate Variability/Change

**Dear respondents,**

This study is carried out by Ahmed Aliyi to collect basic information/ data about agroecological based smallholder farmers’ livelihood vulnerability to climate variability/change in Oromo Nationality administration (ONA). The objective of this questioner is to collect primary data on demographic, socio-economic, environmental, and physical those are required to assess perception of smallholder farmers on climate change, and their vulnerability to climate change induced effects. Therefore, you are kindly requested to give your response freely and accurately to ensure the success of this study.

Dear respondents: you should be confident that the data/information which you give me works only for this study.

Lastly, I thank you for your cooperation!

**Survey information**

1. Address: name of data collector___________________ date___________________ woreda (1= Bati 2= Dawachafa 3= Artuma Fursi 4= Jille Tumuga 5= Dawe Harawa 6= Kemissie Towns 7= Bati Town_______________ kebele (1= Bira 2= Kebele 3= Salate 4= Sitir 5= Lugo 6= Garbi) _________ code (SB, KB, BB, SD, LJ & GJ)____________ study area Oromo Nationality (ONA)
2. Agroecological zone(AEZ): 1. Kolla 2 Woyina Daga 3 Daga

Checked by__________________ signature_________________

**Demographic and socio-economic characteristics**

3. Sex 1= male 2= female

4. Age of the HH head _____________ (in years)

5. Marital status 1= married 2= not married 3= divorce 4= other (specify)______

6. Educational status 1= illiterate 2=capable reading and writing 3=primary school 4=secondary school or above

7. Would you tell me your livelihood source? 1= crop production 2= livestock 3= mixed agriculture 4= government employee 5=trade 6= Hand craft 7= casual lobor 8= other (please specify)__________

8. Family size ________________? Of these how many falls:

1= below 15 years of age (children): 1=male___ 2=female____ 3=total____

2= above 15 and below 65 years of age (adult): 1=male__ 2=female__ 3=total__

3= above 65 years of age (old): 1= male__ 2= female____ 3= total______

9. Do you have your own farm land? 1= Yes 2= No

10. If your answer for question9 is yes, would you tell me your land holding size? ____________

| Types of land use | Area in hectare or Timad |
| --- | --- |
| 1=Cultivated (farm) land |  |
| 6=Irrigated land |  |
| 7=Total land holding |  |

1. Would you tell me about the characteristics of your farm plots (slope situation, and fertility status)?

| Plot Identification | Slope of farm land  1= upper  2= middle  3= lower | Soil fertility status  1= poor  2= moderate  3= fertile |
| --- | --- | --- |
| Farm land |  |  |
| Irrigated |  |  |

**Farmers’ perception on the effects of climate variability**

14. For each of the question below, circle the response that best characterizes how you feel about the statement, where: 1= strongly disagree 2= disagree 3=undecided 4= agree 5= strongly agree

| Perception questions/statement | Strongly disagree | Disagree | Undecided | Agree | Strongly agree |
| --- | --- | --- | --- | --- | --- |
| Climate is changing |  |  |  |  |  |
| There is change in the amount of rainfall |  |  |  |  |  |
| Rainfall is changing |  |  |  |  |  |
| The rainfall amount is decreasing |  |  |  |  |  |
| Too late onset of main season (Kiremt) rainfall |  |  |  |  |  |
| Early cessation of main season rainfall |  |  |  |  |  |
| Temperature of the area is changing |  |  |  |  |  |
| Temperature is increasing |  |  |  |  |  |
| Climate change (CC) can be adapted |  |  |  |  |  |

15. What are the local indicators those show the variability/change in temperature through time in your surrounding?

1= frequency of occurrence of drought and floods

2= human and animals diseases that has not seen before

3= the emergence of new species of animals and plants in your local areas

4= deterioration of rivers through time

5= other (please specify it) ______________________

16. What are the local indicators that show the variability/ change in rainfall through time in your surrounding? ***’Multiple answer is possible’***

1. Changes in crop season
2. Changes in productivity
3. Migration of livestock’s
4. Conflict on natural resources
5. Other, please specify ________________________________

**Vulnerability of small holder farmers to climate variability and change across different agroecological zones (Component of Vulnerability: AC, Exp & Sen)**

**Adaptive capacity characteristics**

**Socio-demographic (1)**

1. Do you live with orphans? 1= Yes 2= No
2. Have you received any training to cope with climate change? 1= Yes 2= No

**Infrastructures (2)**

1. What is the distance to market from your home? _____________ (km or walking hours)
2. What is the distance to all- weather roads from your home_________ (km or walking hours)
3. Do you send your children to school? 1= Yes 2= No
4. What is the distance of your home to the nearest school____________ (km or walking hours)
5. What is the distance to veterinary services from your home?________ (km or walking hours)
6. What is the distance to Health services from your home? _________ (km or walking hours)
7. Do you have access to radio? 1= yes 2= No
8. Do you have access to mobile-phone? 1= yes 2= No
9. Do you have access to climate information? 1= Yes 2= No
10. If your answer for question Q31 is yes, what is your means of information? 1= radio 2= television 3= Agricultural experts 4= other, please specify_____________
11. Do you have access to saving? 1= Yes 2= No
12. If ‘’Yes”, what is your average amount of saving in a year (Birr)? ____
13. Do you have access to credit? 1= Yes 2= No
14. Do you have access to credit? 1= Yes 2= No
15. If ‘’Yes”, How many mount of credit (in Birr) received annually? ________________
16. How many amount of credit (in Birr) received annually in average? ________________

**Housing (3)**

1. Does your house affected by climate related disasters? 1= Yes 2= No

**Technology (4)**

1. What is your production means of farming? 1= Family labor 2= Shared labor 3= Animal traction 4=Tractor 5= others (specify)? ___
2. Do you use farm inputs? 1= Yes 2= No
3. If yes, do you use improved seeds? 1= Yes 2= No
4. If your answer for Q41 is No, why? 1= it is expensive 2= lack of regular supply 3= lack of awareness 4=Others (specify)____________
5. Do you use artificial fertilizers? 1= Yes 2=No
6. If your answer is No, why? 1= lack of access 2= expensive 3= lack of awareness 4= others (specify)?_____
7. Do you use insecticide and pesticides whenever necessary? 1= Yes 2= No
8. Do you use irrigation? 1= Yes 2= No

**Social networks (5)**

1. Are you a member of farmers group in your area? 1= Yes 2= No
2. If yes, which groups do you belongs to? 1= *IDIR* membership 2= *IQUB* membership 3= Famers’ cooperatives Association (FCA) 4= other please specify_________________
3. In the past 12 months, have you or someone in your family gone to government for any assistance? 1= Yes 2= No

**Livelihood strategies (6)**

1. Would you tell us, please, your Gross annual income from non/off farm (salaried job, skilled, labor wage, wage from working on others farm , sales of natural resources (tree, firewood, charcoal) , land rent-out, renting out of agricultural tools )**_____________** (Eth Birr)
2. Would you tell us, please, your Gross annual income from on-farm (agricultural income (crop, livestock, and selling forest products)? **____________** (Eth Birr)
3. Do you have remittance? 1= Yes 2= No

57.If yes, could you tell as Remittance annual income________________

**Asset**

1. How many livestock do you own? ____________

1= Oxen _____ 2= Sheep ____ 3= Cow______

4= Goat _______ 5= Calves ____

6=. Camels _______ 7= Donkey _____

1. What are the main problems you faced in relation to livestock production and productivity?

1= Shortage of feed for animals

2= Animal disease

3= Shortage of open space for keeping

4= Lack of better breeds

5= Lack of veterinary services

6= Shortage of water

7= others (specify) ________________

1. What criteria’s are often used to wealth classification (as poor, medium or rich? 1= number of livestock 2= size of land 3= cash 4= being a leader 5= other specify_____________
2. How do you perceive yourself on wealth classification? 1= poo 2= medium 3= rich

**Food (7)**

1. Where does your family get most of its food? 1= own production 2= purchased 3= aid 4= Others (specify)----------------------
2. Do you have enough food throughout the year? 1=Yes 2= No
3. If No, for how many months you are food insufficient? ____ month (s)

**Health (8)**

1. Is anybody in your family chronically ill (they get sick very often)? 1= Yes 2= No
2. Are you or someone in your family suffering from communicable diseases (disease caused by viruses or bacteria from another person or animal to another like sexual transmitted diseases (STD), HIV/AEDS, Ebola, Flu, and Diarrhea)? 1= Yes 2= No
3. Has anyone in your family been so sick in the past 6 months that they had to miss work or school? 1= Yes 2= No

**Exposure characteristics**

1. **Natural disaster [2= Change in temperature 3= Change in rainfall]**
2. Have you been affected by natural disaster? 1= Yes 2= No
3. If yes, which natural calamities commonly affect your surrounding? 1= drought/flood 2= landslide 3= other, specify______________
4. If your answer for Q75 is flood/drought, how many times has this area been affected by a flood/drought in 1988–2017(for the last 30 years)? _______________
5. If your answer for Q75 is landslide, how many landslide events happened in your surrounding _______________
6. Have you faced crop failure? 1= Yes 2= No
7. If yes, please tell me frequency crop failure in 30 years (1987-2016) _____________
8. Was anyone in your family injured in the flood/drought? 1= Yes 2= No
9. Did anyone in your family die during the flood/drought? 1= Yes 2= No
10. Is anybody in your family is with no jobs during those extreme events happened? 1= Yes 2= No
11. Do you have access to early-warning about natural disaster (flood/drought) before happening of the event? 1=Yes 2=No

**Sensitivity characteristics**

**Water (1)**

1. Do you have access/availability to clean water? 1= Yes 2= No
2. What is the distance to water source from your home?____________ (km or distance hours)
3. Do you have a consistent water supply? 1= Yes 2= No
4. Do you have training on farm management? 1= Yes 2=No
5. What is your number one annual crop production you are producing in a year?

| Type of crop production | Products (in Quintals or kg) |
| --- | --- |
| 1.Sorghum |  |

1. Is there change in your annual crop yield per hectare? 1= yes 2= No
2. If yes, what kind of change is it? 1= decreasing 2= increasing 3= I don’t know 4= other, specify __________
3. If your answer is decreasing what is the change (productivity in Quintals/hectare)? _______
4. If your answer is increasing, how many of quintals/hectare is increasing? ______
5. Do you diversify your crop? 1= Yes 2=No
6. Does your family save seeds? 1= yes 2= No

**Natural resources (3)**

1. Does your family exploit natural resources? 1= yes 2= No
2. What are the main energy sources for cooking your food? 1= Electricity 2= Charcoal only 3= Gas 4= Firewood only 5= Crop residue 6= other specify______________
3. Do you think firewood is changing in amount in the past 30 years? 1= Yes 2= No
4. If yes, what change do you observe? 1= Increasing 2= Decreasing 3= I don’t know 4= other (please specify)__________________

**Soil and water (4)**

1. Is their land degradation by climate change? 1= yes 2= No
