# Supplementary material for: Vulnerability of smallholder farmers to climate variability and change across different agro-ecological Zones in Oromo Nationality Administration (ONA), North east Ethiopia
Source: PLoS One. 2022 Jun 22;17(6):e0268094. doi: 10.1371/journal.pone.0268094 (PMC9216564; doi:10.1371/journal.pone.0268094)
Supplement: Attachment — Submitted filename: Review Note.pdf [file pone.0268094.s003.pdf]

The study tried to assess vulnerability of smallholder farmers to climate variability and change across different agro-ecological zones in Oromo Nationality Administration (ONA), North east of Ethiopia. I would like to recommend the authors to improve the manuscript considering the following comments.

1- What is the novelty of study? There is a body of literature conducted to assess vulnerability of smallholder farmers to climate variability based on agro-ecological system approach, over Ethiopia (e.g. Asfaw et al,. 2021, Gebru at al., 2020, Mohammed et al., 2021, Zeleke at al., 2021) and a few of the research works have been also cited in manuscript (ref. number: 7,8, 23 & 24). What is the particular problem or approach that nobody else tried for it yet? The authors claim "little studies used expert judgment approach in weighting indicators of vulnerability components. Hence, this study aimed to fill the gap in methodology." However, the authors did not also provide a novel method for their analysis, and simply applied the methods commonly employed in the published works.
2- In addition to using only expert judgment for assigning weight to indicators, the study could use statistical method (Principal Component Analysis (PCA) or Factor Analysis (FA)) or the arbitrary choice of equal weight. Though methods have been extensively applied in the literature, the comparison can add new findings to the existing literature.
3- This study appears to be site-specific and can be more considered as a case study which may be interesting for local communities but not for international audiences. Moreover, it is difficult for readers to understand and follow information under LVI results section. Therefore, it is helpful to define levels of vulnerability to each agro-ecology. Authors are suggested to consider vulnerability scores to categorize vulnerability across the study area with low vulnerable, moderate and high vulnerable levels.
4- The manuscript needs a round of proofreading. Many sentences should be completed by dot after reference number. Grammar and English writing need to be improved. For example "Little studies" should be revised into "few studies".
5- The other concern is related to some apparent missing in manuscript. It is suggested that authors review the manuscript carefully. Examples are as below:

a) Where is the parameter "n" in equation (1)? (It should be clear for reader that equation (1) is equal to n which is the total sample size for the study). Moreover, a part of Equation 1; (N-2), differs from the calculation four lines after (6347-1), and It seems this part should be edited to (N-1) in the equation as below:

$$\boldsymbol{n} = \frac{\boldsymbol{z^2 * p * q * N}}{\boldsymbol{e^2(N-1) + Z^2 * p * q}}$$

b) Reference 24: the referencing is wrong, it seems that the authors mixed two following reference together!

Chala, D., Belay, S., Bamlaku, A., & Azadi, H. (2016). Agro-ecological based small-holder farmer's livelihoods vulnerability to climate variability and change in Didesa sub Basin of Blue Nile River, Ethiopia. Academia Journal of Agricultural Research, 4(5), 230-240.

Dechassa, C., Simane, B., & Alamirew, B. (2017). Farmers' livelihoods vulnerability to climate variability and change in Didesa Basin southern part of Abay Basin, Ethiopia. In Climate Change Adaptation in Africa (pp. 267-284). Springer, Cham.

c) In addition to "Livelihood Vulnerability Index (LVI)" Authors should make it clear for reader that "LVI-IPCC" is abbreviation for LVI-Intergovernmental Panel on Climate Change.

References in review:

- Asfaw, A., Bantider, A., Simane, B., & Hassen, A. (2021). Smallholder farmers' livelihood vulnerability to climate change-induced hazards: agroecology-based comparative analysis in Northcentral Ethiopia (Woleka Sub-basin). Heliyon, 7(4), e06761.
- Gebru, G. W., Ichoku, H. E., & Phil-Eze, P. O. (2020). Determinants of smallholder farmers' adoption of adaptation strategies to climate change in Eastern Tigray National Regional State of Ethiopia. *Heliyon*, *6*(7), e04356.
- Mohammed, Y., Tesfaye, K., Tadesse, M., & Yimer, F. (2021). Analysis of smallholder farmers' vulnerability to climate change and variability in south Wollo, north east highlands of Ethiopia: An agro-ecological system-based approach.
- Zeleke, T., Beyene, F., Deressa, T., Yousuf, J., & Kebede, T. (2021). Vulnerability of Smallholder Farmers to Climate Change-Induced Shocks in East Hararghe Zone, Ethiopia. Sustainability 2021, 13, 2162.
