# Supplementary material for: Vulnerability of smallholder farmers to climate variability and change across different agro-ecological Zones in Oromo Nationality Administration (ONA), North east Ethiopia
Source: PLoS One. 2022 Jun 22;17(6):e0268094. doi: 10.1371/journal.pone.0268094 (PMC9216564; doi:10.1371/journal.pone.0268094)
Supplement: Attachment — Submitted filename: PONE-D-21-29744_Reviewer_comments..docx [file pone.0268094.s004.docx]

**Manuscript ID: PONE-D-21-29744**

**General comments:**

The manuscript entitled " Vulnerability of small holder farmers to climate variability and change across different agro-ecological Zones in Oromo Nationality Administration (ONA), Northeast Ethiopia" by Ahmed Aliy Ebrahim et al. examined the agro-ecological based smallholder farmer’s livelihood vulnerability to climate variability and change. The topic is of general interest to the readers of the journal. Several tables and figures have appropriately been used to present the findings. I have some suggestions for authors, which are listed as follows.

1) Justification of the study should be more elaborated. Why was this area selected as the study location? Why only smallholder farmers had been considered? - these issues should be addressed clearly.
2) Methodology should be more explained. Especially, variables/indicators used in the study could be summarized in a table with adequate information.
3) Justification of indicator/variable selection along with references could improve this section more.
4) Why only 3 indicators were considered for ‘Technology’ component? - Explain with reference.
5) Why only three indicators were considered for Asset? How you have measured the ‘fertile soil of the farm land’? Please explain with necessary references. Try to compare the findings with previous studies.
6) How ‘enough food for nutrition’ mentioned under ‘Food’ sub-section in result and discussion section, was measured? It should be mentioned in methodology. Compare the result of this sub-section with previous studies. Please make some discussions based on your findings instead of just describing the results.
7) ‘Health’ sub-section under result and discussion part should be re-written by making it more clear to the readers.
8) In case of frequency of flood and drought, you are mentioning in text about last 10 years but in parenthesis you have written 2011-2016. Please make correction.
9) In case of climate variability change, it would be better to include secondary climate data and compare with farmers perception.
10) Please compare the findings of ‘Water’ sub-section with previous findings.
11) What are the reasons behind the perception of decrease in crop yield mentioned in the ‘Agriculture’ sub-section, please mention?
12) More effort should be made on the implication of the study findings in ‘result and discussion’ section.
13) For indicating the citation in the main text, it is appropriate to use […] instead of (…).
14) There are some typo throughout the manuscript. Please go through carefully and make corrections.
