# Supplementary material for: Vulnerability of smallholder farmers to climate variability and change across different agro-ecological Zones in Oromo Nationality Administration (ONA), North east Ethiopia
Source: PLoS One. 2022 Jun 22;17(6):e0268094. doi: 10.1371/journal.pone.0268094 (PMC9216564; doi:10.1371/journal.pone.0268094)
Supplement: Attachment — Submitted filename: Manuscript ID PONE-D-21-29744R1_RC.docx [file pone.0268094.s006.docx]

**Manuscript ID: PONE-D-21-29744R1**

The manuscript entitled " Vulnerability of small holder farmers to climate variability and change across different agro-ecological Zones in Oromo Nationality Administration (ONA), Northeast Ethiopia" by Ahmed Aliy Ebrahim et al. examined the agro-ecological based smallholder farmer’s livelihood vulnerability to climate variability and change. The topic is of general interest to the readers of the journal. Several tables and figures have appropriately been used to present the findings.

All my comments have been fulfilled by the authors. As a result, I recommend that this work be published.
