# Supplementary material for: Vulnerability of smallholder farmers to climate variability and change across different agro-ecological Zones in Oromo Nationality Administration (ONA), North east Ethiopia
Source: PLoS One. 2022 Jun 22;17(6):e0268094. doi: 10.1371/journal.pone.0268094 (PMC9216564; doi:10.1371/journal.pone.0268094)
Supplement: Attachment — Submitted filename: Response to reviewers.doc [file pone.0268094.s007.doc]

Dear, Shamsuddin Shahid
Academic Editor
PLOS ONE

Subject: Ref.: No. PONE-D-21-29744R1

We are writing this with reference to the revised manuscript entitled Vulnerability of small holder farmers to climate variability and change across different agro-ecological Zones in Oromo Nationality Administration (ONA), North east Ethiopia, which has be submitted for publication in PLOS ONE. We would like to express our sincere gratitude to the reviewers for their valuable comments and suggestions. We have revised the manuscript by taking into account the comments and suggestions given by reviewers (Reviewers #1 and #2). We are now re-submitting the revised version for your kind reconsideration for publication. We sincerely hope that we have sufficiently addressed the suggested comments, and have prepared the manuscript in accordance with the journal’s style. The details of how the comments and suggestions as addressed point by point are given below.

Looking forward to hear your positive response to publish our manuscript in your Journal,

=====

**Rebuttal letter**

**Response to the Journal Requirements**

Please review your reference list to ensure that it is complete and correct. If you have cited papers that have been retracted, please include the rationale for doing so in the manuscript text, or remove these references and replace them with relevant current references. Any changes to the reference list should be mentioned in the rebuttal letter that accompanies your revised manuscript. If you need to cite a retracted article, indicate the article’s retracted status in the References list and also include a citation and full reference for the retraction notice.

[Note: HTML markup is below. Please do not edit.]

- **Response: The reference list is complete and correct.**

Reviewers' comments:

Reviewer's Responses to Questions

**Comments to the Author**

1. If the authors have adequately addressed your comments raised in a previous round of review and you feel that this manuscript is now acceptable for publication, you may indicate that here to bypass the “Comments to the Author” section, enter your conflict of interest statement in the “Confidential to Editor” section, and submit your "Accept" recommendation.

Reviewer #1: (No Response)

Reviewer #2: All comments have been addressed

- **Response: Thank you very much**

2. Is the manuscript technically sound, and do the data support the conclusions?

The manuscript must describe a technically sound piece of scientific research with data that supports the conclusions. Experiments must have been conducted rigorously, with appropriate controls, replication, and sample sizes. The conclusions must be drawn appropriately based on the data presented.

Reviewer #1: Yes

Reviewer #2: Yes

- **Response: Thank you very much**

3. Has the statistical analysis been performed appropriately and rigorously?

Reviewer #1: N/A

Reviewer #2: Yes

- **Response: Thank you very much for your comments.**

4. Have the authors made all data underlying the findings in their manuscript fully available?

The [PLOS Data policy](http://www.plosone.org/static/policies.action#sharing) requires authors to make all data underlying the findings described in their manuscript fully available without restriction, with rare exception (please refer to the Data Availability Statement in the manuscript PDF file). The data should be provided as part of the manuscript or its supporting information, or deposited to a public repository. For example, in addition to summary statistics, the data points behind means, medians and variance measures should be available. If there are restrictions on publicly sharing data—e.g. participant privacy or use of data from a third party—those must be specified.

Reviewer #1: Yes

Reviewer #2: Yes

- **Response: Thank you very much**

5. Is the manuscript presented in an intelligible fashion and written in standard English?

PLOS ONE does not copyedit accepted manuscripts, so the language in submitted articles must be clear, correct, and unambiguous. Any typographical or grammatical errors should be corrected at revision, so please note any specific errors here.

Reviewer #1: Yes

Reviewer #2: Yes

- **Response: Thank you very much for your comments.**

6. Review Comments to the Author

Please use the space provided to explain your answers to the questions above. You may also include additional comments for the author, including concerns about dual publication, research ethics, or publication ethics. (Please upload your review as an attachment if it exceeds 20,000 characters)

Reviewer #1: The authors have adequately addressed the comments raised in the first round of review. The manuscript is more coherent now and the quality of the revised manuscript has been improved. Overall, the paper is well written and sounds technically good.

- **Response: Thank you very much for your positive response**

There are minor comments before considering for publication.
Abstract, Line 53: It is suggested to avoid using the personal form (we conclude …). Passive voice and third person form should be used for the whole manuscript.

- **Response: Fixed. Thank you very much for your comments.**

Keywords, agro-ecology: Please capitalize first word

**Response: Fixed.**

Introduction, line 112: “As a result, this paper aimed to examine agro-ecological based…”
&
Conclusion, line 667: “This study examines agro ecological based ...”,
Please be consistent for using hyphen over the whole paper and it looks it can be edited as “… agro-ecological zones…” to better conveying of meaning.

- **Response: Fixed. Thank you very much for your comments.**

Reviewer #2: (No Response)

7. PLOS authors have the option to publish the peer review history of their article ([what does this mean?](https://journals.plos.org/plosone/s/editorial-and-peer-review-process#loc-peer-review-history)). If published, this will include your full peer review and any attached files.


If you choose “no”, your identity will remain anonymous but your review may still be made public.


**Do you want your identity to be public for this peer review?** For information about this choice, including consent withdrawal, please see our [Privacy Policy](https://www.plos.org/privacy-policy).

Reviewer #1: No

Reviewer #2: **Yes:** Mohammad Kamruzzaman, Farm Machinery and Postharvest Technology Division, Bangladesh Rice Research Institute, Gazipur-1701, Bangladesh, email: milonbrri@gmail.com

[NOTE: If reviewer comments were submitted as an attachment file, they will be attached to this email and accessible via the submission site. Please log into your account, locate the manuscript record, and check for the action link "View Attachments". If this link does not appear, there are no attachment files.]

While revising your submission, please upload your figure files to the Preflight Analysis and Conversion Engine (PACE) digital diagnostic tool, <https://pacev2.apexcovantage.com/>. PACE helps ensure that figures meet PLOS requirements. To use PACE, you must first register as a user. Registration is free. Then, login and navigate to the UPLOAD tab, where you will find detailed instructions on how to use the tool. If you encounter any issues or have any questions when using PACE, please email PLOS at [figures@plos.org](mailto:figures@plos.org). Please note that Supporting Information files do not need this step.

*In compliance with data protection regulations, you may request that we remove your personal registration details at any time.* [*(Remove my information/details)*](https://www.editorialmanager.com/pone/login.asp?a=r)*. Please contact the publication office if you have any questions.*
